# Bibliography

**Published:** 1891-12

**Authors:** 


### Bibliography.

---

THE POCKET MATERIA MEDICA AND THERAPEUTICS. By C. HENRI LEONARD, A.M., M.D. The Illustrated Medical Journal Co., Detroit, Mich., 1891.

This book, "designed for the practitioner as much as for the student," has been compiled with much care. "The newer remedies are not to be found in the dispensatories, pharmacopœias, or standard works upon materia medica," hence it combines much valuable matter not obtainable except by laborious search through various sources of information.

It will be found a convenient volume for the physician, student, or druggist.

DENTAL MEDICINE: A MANUAL OF DENTAL MATERIA MEDICA AND THERAPEUTICS. By FERDINAND J. S. GORGAS, M.D., D.D.S. Fourth Edition, Revised and Enlarged. P. Blakiston, Son & Co. Philadelphia, 1891.

This standard work of dental materia medica has been in such demand that each edition has become exhausted before the completion of that succeeding. This rapid sale is indicative of the appreciation felt for it in college circles. It has met a want as a textbook, and has become a necessity to the dental student and to the practitioner who wishes to keep himself informed without the labor of examination of larger and more original works. Much new matter has been added, bringing the book to five hundred and twenty-four pages. Condensation will be required in future editions, as this has already reached the limit of a convenient textbook.

In addition to matter on various subjects, the following list of "new antiseptics, disinfectants, germicides, hypnotics, etc., will be found fully considered: Aristol, bromol, campho-phenique, phenol-camphor, chloralamide, synthetic carbolic acid, biniodide of mercury, iodine, trichloride, chloral-phenol, iodo-phenacetin, lysol, bichloride of methylene, microcidine, myrtol, phenacetin, pyoktanin, salol, sodium, silico-fluoride, salipyrene, etc.

The opening of a work of this kind, with fifty-six pages descriptive of pathological conditions, seems altogether inappropriate. While this matter has a value apart from its present connections, it might be omitted in future editions without detriment to the main subject.

The author continues the paragraph in regard to the application of arsenic on sensitive dentine, notwithstanding repeated criticisms made on the former editions. He writes: "As it (arsenic) is capable of being absorbed through a considerable thickness of dentine, the result of which would be the death of the pulp, arsenious acid, if it is employed for obtunding the sensibility of the dentine, *should be suffered to remain in the tooth but a very short time,—from one to three hours,—and every particle of it carefully removed.*" (Italics ours.)

Three hours would be amply sufficient to carry the destructive process beyond reparation. Exactly how *every particle of arsenious acid* is to be removed after three hours is not explained.

The habit of this writer still is evident of appropriating freely from the original work of others and weaving it in the text. Criticism has stimulated to a show of fairness in this direction, but there is still much room for improvement. The ordinary facts in materia medica are generally public property, but even here there is a limit to appropriation without credit; but when dental matters of a practical bearing are considered, care should be taken to designate the originator of a special process.

The attention of the author is called to page 163, to bad proof-reading. There is no such person as Charles Truman, nor did such an individual write on iodoform and arsenic as a "painless devitalizer under all conditions." The article in question was written by James Truman.

The author has been assiduous in his efforts to bring into book form all the agents of any value in materia medica recently introduced. The dental student will, therefore, find nothing lacking to aid him in the study of this rapidly-growing subject.

Annual of the Universal Medical Sciences: A Yearly Report of the Progress of the General Sanitary Sciences throughout the World. Edited by Charles E. Sajous, M.D., and Seventy Associate Editors. Illustrated with Chromo-Lithographs, Engravings, and Maps. F. A. Davis, Publisher. Philadelphia, New York, Chicago, Atlanta, and London, 1891.

This well-known publication, edited by Dr. Sajous and his associates, is again presented to the medical world in five volumes.

This unique work has been completed for 1891 most satisfactorily and under many difficulties. It is questionable whether any but those directly engaged can fully appreciate the gigantic character of this undertaking,—sufficient to appall any ordinary man; but the editor-in-chief is not an ordinary worker, as the volumes published abundantly prove.

The list of associate editors accompanying each volume is a guarantee as to the thoroughness of preparation of each department, making it truly a universal synopsis of medical progress during the year throughout the world. To this is added a full index and reference list of publications quoted, to which page and date are supplied in the text. This is a valuable addition.

This is a period in the history of labor when the world's knowledge requires condensation. The progressive mind in all departments of culture is so active that the average man cannot keep pace with it, much less can he afford the time to search for the facts so necessary in practice, and which are required to render him intelligent on all subjects. If this be true of mankind in general it has special application to the medical and dental practitioner. It was, therefore, eminently proper that America should produce this publication, as it is here more than anywhere else that concentration finds its greatest necessity. These volumes fulfil this requirement most satisfactorily.

It is to be regretted that dental science finds no place in this publication. This is recognized as no fault of the editor, as every effort was made to secure co-operation of the dental profession in this direction.

If they are not represented in its pages, they should, at least, estimate the great value of the labors of those who have done this work by adding it to their libraries, year by year, as it appears laden with the fresh fruits of the busy brains and hands of investigators in medical science everywhere.

A COMPEND OF HUMAN PHYSIOLOGY, ESPECIALLY ADAPTED FOR THE USE OF MEDICAL STUDENTS. By ALBERT P. BRUBAKER, A.M., M.D. Sixth Edition, Revised and Improved. P. Blakiston, Son & Co. Philadelphia, 1891.

The Quiz Compends issued by this firm have a positive value as books of reference, and this by Dr. Brubaker is no exception. The fact that it is in its sixth edition is proof sufficient that it has been appreciated by those for whom it was intended.

Condensation is an art not always attained by those who wish to impart information. To express ideas in words which will convey the entire subject, omitting nothing of vital importance, is difficult. In this Dr. Brubaker excels, and his compend not only covers the information demanded for the student but it can be read with interest by those more advanced.

THREE THOUSAND QUESTIONS ON MEDICAL SUBJECTS, ARRANGED FOR SELF-EXAMINATION. P. Blakiston, Son & Co. Philadelphia, 1891.

This little book, prepared for medical students evidently by a teacher of experience, has a positive value. Every student is familiar with the fact that a self-examination involving constant repetitions is the only means to impress any subject upon the individual, but this is not always possible without outside aid.

A correct system of reference is adopted by which the "student with the least expenditure of time can look up the subject and thus doubly fasten it firmly on the mind." As an aid to study, this convenient book can be recommended without any reservations.

THE VEST-POCKET ANATOMIST. (Founded upon Gray.) By C. HENRI LEONARD, A.M., M.D. Fourteenth Revised Edition. The Illustrated Medical Journal Co., Detroit, 1891.

This well-known compend, and, as its name implies, no larger than can be conveniently carried in the pocket,—not the vest-pocket, however,—is presented again in another edition to professional readers.

As it is founded on Gray, with one hundred and ninety-three illustrations, it becomes a convenient book of reference to students; and while it does not exclude or replace the larger work, it will be found a most excellent substitute, and one always in place to refresh the memory or to direct dissection.

